# Metabolism and Tissue Distribution of Chelerythrine and Effects of *Macleaya Cordata* Extracts on Liver NAD(P)H Quinone Oxidoreductase

**DOI:** 10.3389/fvets.2021.659771

**Published:** 2021-05-26

**Authors:** Chong-Yin Huang, Ya-Jun Huang, Zhuo-Yi Zhang, Yi-Song Liu, Zhao-Ying Liu

**Affiliations:** ^1^College of Veterinary Medicine, Hunan Agricultural University, Changsha, China; ^2^Hunan Engineering Technology Research Center of Veterinary Drugs, Hunan Agricultural University, Changsha, China; ^3^Hunan Prima Drug Research Center Co., Ltd., Changsha, China; ^4^Yiyang Vocational and Technical College, Yiyang, China

**Keywords:** *Macleaya cordata*, sanguinarine, chelerythrine, HPLC/QqTOF-MS, metabolites, reduction

## Abstract

**Background:** *Macleaya cordata (Willd.) (Papaveraceae)* is listed as a feed additive in animal production by the European Food Authority.

**Methods:** The metabolites of chelerythrine in rats were measured *in vitro and in vivo* by rapid and accurate high-performance liquid chromatography/quadrupole-time-of-flight mass spectrometry (HPLC/QqTOF-MS). The structures of CHE metabolites were elucidated by comparing their changes in accurate molecular masses and fragment ions with those of parent ion or metabolite. The metabolic enzymes that were involved in chelerythrine reduction were investigated using an inhibition method. The tissue distribution of chelerythrine and the effects on NQO1 following intragastric administration with *M. cordata* extracts in rats were examined.

**Results:** A total of twelve metabolites of chelerythrine were characterized by this approach in rat liver S9 and *in vivo*. The reduction of the iminium bond of chelerythrine and subsequent O-demethylation was the main metabolic pathway of chelerythrine in rat liver S9 while the reduction of the iminium bond of chelerythrine was the main metabolic pathway of chelerythrine in rats *in vivo*. After the rats were given intragastric administration, the low concentration residues of sanguinarine and chelerythrine in different rat tissues were found at 48 h after the last dose, suggesting that both compounds could be widely distributed in tissues. The results also indicated that XO, NQO1, NQO2, and carbonyl reductase are involved in chelerythrine reduction. *Macleaya cordata* extracts treated female and male rats, respectively, showed different responses, inhibiting NQO1 activity in males, but inducing NQO1 activity in females. Chelerythrine had a weak impact on NQO1 activity, but sanguinarine inhibited NQO1 activity

**Conclusion:** Through studying the effects of cytosolic reductase inhibitors on chelerythrine reduction and the impact of chelerythrine and sanguinarine on the activity of NQO1 *in vitro* and *in vivo*, we clarified the potential drug interaction of *Macleaya cordata* extract in clinical application, so as to provide theoretical guidance for clinically safe medication. In addition, it provided a reference basis for the metabolic mechanism of chelerythrinein rats.

## Introduction

*Macleaya cordata (Willd.) R.Br. (Papaveraceae)*, a perennial plant, is listed as feed additive for animal production by the European Food Safety Authorityin 2004 (1). The powdered mixture of seeds, capsules, and leaves are the main components of the feed additive Sangrovit (2). The main active components of *M. cordata*are are Sanguinarine and chelerythrine (3). Quaternary benzo[c]phenanthrine alkaloids (QBAs) can improve the performance of an animal by eliminating the demand for antibiotics in the feed (3). Moreover, sanguiritrin in *M. cordata* is made up of QBAs in part containing sanguinarine and chelerythrine, which has been used as an antiplaque agent for mouthwash and toothpaste, and in veterinary preparation for the treatment of mastoiditis in cows. The efficacy and safety of sanguiritrinin oral care has been tested in many long-term and short-term clinical trials, e.g., using products presented by Paraskevas (4). Sanguiritrin was reportedly authorized by the Ministry of Health of the Union of Soviet Socialist Republics for industrial production and a wide range of medical uses for pharmaceutical formulations. In 1982, the pharmaceutical industry began to produce the drug (5). The extracts or compounds of *M. cordata* have significant pharmacological activities, but they are also toxic. Thus, the pharmacokinetic study of chelerythrine in animals is very important due to its biological effects and commercial use.

Previous pharmacokinetic studies have indicated that the bioavailability of chelerythrine was apparently low after oral administration. Kosina and their research group investigated the levels of chelerythrine in plasma, tissues and feces of pigs (6). The results showed that the chelerythrine was mostly excreted in feces and a small proportion of alkaloid was absorbed. Some *in vitro* metabolism studies also indicated that formation of the dihydrochelerythrine (DHCHE), which may be accompanied by special processes of O-demethylenation/O-demethylation, was the main mechanism behind the biotransformation of chelerythrine (7–9). Using the HPLC/QqTOF-MS method, Vacek et al. analyzed and then suggested the putative structures of 11 phase I and 5 phase II metabolites of chelerythrine using human hepatocytes (9). However, the *in vivo* metabolic profiles of chelerythrine have not yet been systematically elucidated. Detailed structural description of chelerythrine metabolites based on their product ions is rarely reported. Therefore, it is important to obtain more detailed descriptions of the metabolism of chelerythrine *in vivo*.

Chelerythrine and sanguinarine can be reabsorbed by QBA-producing plant cells and reduced to the less toxic dihydro derivativesm by sanguinarine reductase (10). Also, in animals and humans, the first step in the biotransformation of chelerythrine and sanguinarine appears to be transforming into their corresponding dihydro derivatives. Some studies have demonstrated that sanguinarine can be reduced to dihydrosanguinarine (DHSA) in the presence of enzyme cofactors NADH and NADPH (11, 12). In addition, our research team has demonstrated that in the liver cytosol, the reduction of the sanguinarine iminium bond occurs in two ways. One way is enzymatic reduction by possible carbonyl and/or quinone reductases in the hepatic cytosol, and the other is direct non-enzymatic reduction by NAD(P)H (13). We have also elucidated that the NQO1 was involved in the iminium bond reduction of sanguinarine, which in turn decreased cytotoxicity and sanguinarine-induced apoptosis (14). Numerous published studies have demonstrated that the main metabolic pathway of SA and CHE is reduction in the iminium bond, resulting in the formation of DHSA and DHCHE (7, 15, 16). Some studies have shown that the SA could be quickly metabolized into DHSA by iminium bond reduction which proceeds via two routes in the liver (13, 17). The NQO1 enzyme's involvement in the chelerythrine reduction, based on the similar structure of sanguinarine, requires further studies. As far as we know, there are no reports to evaluate the effects of chelerythrine and sanguinarine on NQO1.

Therefore, the first aim of this study was to investigate the metabolism of chelerythrine *in vitro* and *in vivo*. The second aim of the present study was to examine the metabolic enzymes that are involved in chelerythrine reduction using inhibition methods and effects on NQO1 activity *in vitro*. The third aim was to explore the tissue distribution residues in liver, heart, spleen, lung, and kidney, and the effects on NQO1 following intragastric administration with *M. cordata* extracts (containing 40% sanguinarine and 20% chelerythrine) for 3 weeks in rats.

## Materials and Methods

### Chemicals

Chelerythrine (Content ≥ 95%), sanguinarine (Content ≥ 95%), and *M. cordata* extracts (containing 40% sanguinarine, 20% chelerythrine and residual components include polysaccharides and amino acids) were obtained from Hunan Meikeda Co. (Changsha, China). Reduced metabolites DHCHE and DHSA were prepared using chelerythrine and sanguinarine reduction with NaBH_4_ in methanol, respectively. Deionized water was purified using a Milli-Q system (Bedford, MA, USA). NADPH and NADH were purchased from Roche Chemical Co. (Beijing, China). 7-Hydroxycoumarinand menadione were purchased from Sigma Chemical Co. (Content > 98%, St. Louis, USA). Quercetin (Content ≥ 97%) and dicoumarol (HPLC ≥ 95%) were purchased from the National Institutes for Food and Drug Control (Beijing, China). HPLC-grade acetonitrile and formic acid were purchased from Merck (Darmstadt, Germany) and ROE (Newark, DE, USA), respectively. All other chemicals and reagents used were of the highest analytical grade.

### Metabolism of Chelerythrine in Rat Liver S9

The rat liver S9 was prepared according to our previous metabolism study (18). To investigate the metabolites of chelerythrine *in vitro*, 2 mg/mL rat liver S9 protein, 5 mM MgCl_2_, and 2 mM NADPH were mixed in a final volume of 200 μL of 0.05 M Tris–HCl buffer (pH 7.4). Chelerythrine was dissolved in methanol (final amount in the reaction medium was 1%). The reaction mixture was preincubated at 37°C for 5 min, and then 20 μg/mL chelerythrine was added to initiate the reaction. The incubation mixtures without chelerythrine or NADPH were used as the blank control. 200 μL ice-cold acetonitrile was added to stop the reaction after 1 h. The mixture was vortexed and centrifuged at 12,000 g for 15 min at the end of the reaction. The clear supernatant was analyzed using HPLC/QqTOF-MS to identify metabolites.

### The Chelerythrine Iminium Bond Reduction by Rat Liver Cytosol

The preparation of rat liver cytosol was isolated and incubated with chelerythrine according to our previous sanguinarine report (13). In brief, the incubation mixture consisted of 0.25 mg/mL cytosol protein, 5 mM MgCl_2_, 100 μmol/L inhibitor, and 2 mM NADPH in 0.05 M Tris-HCl buffer (pH 7.4). The inhibitors were dicoumarol (quinone oxidoreductase 1), quercetin (quinone oxidoreductase 2, carbonyl reductase), menadione (aldehyde oxidase, carbonyl reductase), and 7-hydroxycoumarin (xanthine oxidase). The reaction was preincubated at 37°C for 5 min. After the addition of 10 μg/mL chelerythrine, the reaction was started. The incubation mixtures without the inhibitor or cytosol protein acted as the blank control. The reaction was stopped by adding 200 μL ice-cold acetonitrile after 15 min, and the mixture was vortexed and centrifuged at 12,000 g for 15 min. The clear supernatant was analyzed by HPLC for the quantitative results of DHCHE.

### Animals, Drug Administration, and Sample Collection

Sprague-Dawley (SD) rats were purchased from Hunan SLRC Laboratory Animal Center (Changsha, China). The rats were acclimatized for 5 days under a standardized light (12 h light/12 h dark) and temperature (26°C) environment in Hunan Research Center of Laboratory Animals (Changsha, China), and ate standard feed freely. All animal care and experimental protocols were conducted in accordance with the Guide for the Care and Use of Hunan Provincial Laboratory Animal Public Service Center and approved by the Institutional animal care and use committee of Hunan Research Center for drug safety evaluation, Changsha, China (permit number SYXK 2010-0005).

#### In vivo Metabolism

Ten SD rats (160–180 g, five males and five females) were individually placed in stainless steel metabolic cages. All rats were fasted for 12 h and had free access to water before drug administration. Chelerythrine in water with the dissolving aid of Tween-80 was intragastrically administered to rats at a dose of 10 mg/kg. One milliliter whole blood samples were collected from retro orbital sinus with a heparinized blood collection tube at 3 h following drug dosing. Each collected blood sample was centrifuged at 3,000 rpm at 4°C for 10 min to obtain plasma samples. Urine and feces of rats were collected during 0–12 and 12–24 h after administration, respectively. All samples were collected from female and male rats and stored at −70°C.

#### Tissue Distribution

Tissue distribution was carried out in eighteen SD rats (160–180 g, nine males and nine females). Twelve rats (six male and six female) were intragastrically administered with *M. cordata* extracts at a single dosage of 5 mg/kg (containing about 2 mg/kg dose of sanguinarine and 1 mg/kg dose of chelerythrine) in water with the dissolving aid of Tween-80 for 3 weeks. Three male and three female rats were given the same amount of water as the blank control group by intragastric administration. Six rats (three male and three female) in the control group and six rats (three male and three female) in the drug group were sacrificed at 24 h after last dosing, and the rest of the rats were sacrificed by cervical dislocation at 48 h after the administration. Tissue samples including cecal contents, lung, heart, liver, kidney, and spleen were collected at 24 and 48 h after the last administration and stored at −70°C.

#### Sample Pretreatment

The preparations of plasma, urine, feces, and tissues were pretreatments according to our current study (19).

### Instruments and Analytical Conditions

#### HPLC/QqTOF-MS

The chromatographic separation was conducted using an Agilent 1290 HPLC system with a Hypersil GOLD column (150 × 2.1 mm I.D.; particle size 5 μm). The mobile phase was composed of 0.1% formic acid water (mobile phase A) and acetonitrile (mobile phase B), and the gradient elution procedure was as follows: 10% B (0–5 min), 10–90% B (5–20 min), 90% B (20–25 min), 10% B (25–30 min), and the flow rate of 0.3 mL/min. The injection volume was 5 μL.

The Agilent 6530 Q-TOF-MS mass spectrometer equipped with electro-spray ionization (ESI) source operated in positive ionization mode. Nitrogen was used as atomization gas, and the flow rate was 9 L/min. The parameters of the mass spectrometer were as follows: nozzle voltage 1 kv, capillary voltage 4.0 kv, sheath gas temperature 350°C, sheath gas flow rate 11 L/min, nebulizer voltage 35 psig. The quarantine window was set at 1 amu. The instrument automatically performed the internal mass calibration though the automated calibrate transfer system. In positive ion mode, the calibrating solution contained the internal reference masses at m/z 121.0508 and 922.0098. All the data acquisition was controlled by Agilent Mass Hunter software (version B.01.03 Build 1.3.157.0 2), and the Agilent Mass Hunter software (Qualitative Analysis B.04.0) was used to accurately take mass measurements of each peak from the total ion chromatograms. Then, these metabolites were subjected to accurate EIC, and the acceptance or rejection was decided according to the peak appearing in the EIC. Structural elucidations of chelerythrine metabolites were carried out on the basis of the mass change from the metabolite or parent drug, the predicted elemental compositions, and the interpretation of accurate MS/MS spectra.

#### HPLC-MS/MS

The HPLC-MSMS method was established for the determination of sanguinarine and chelerythrine in tissues using the Agilent series 1290 Infinity HPLC system with a 1.8 μmAgilent Zorbax SB-C_18_ (2.1 × 50 mm) column. The mobile phase consisted of 0.1% formic acid water (mobile phase A) and acetonitrile (mobile phase B), and the linear gradient at the flow rate of 0.3 mL/min was as follows: 0–4 min, 15–17%B; 4–6 min, 17–35% B; 6–8 min, 35%B. The injection volume was 2 μL. An Agilent 6460 QQQ mass spectrometer equipped with ESI was operated in positive mode with multiple reactions monitoring mode. The controls of the operating parameters were as follows: capillary voltage, 3,500 V; EM voltage, 200 V; dry gas (N_2_) flow rate, 12 L/min; ion source temperature, 350°C; nebulizer, 25 psi. The quantitative transitions, collision energy (CE) and cone-voltage for sanguinarine were 332.1 > 274.0, 34 eV and 141 V, respectively. The quantitative transitions, CE and cone-voltage for ALL were, 348.1 > 332.0, 30 eV and 146 V, respectively.

Using optimal conditions, the retention times of sanguinarine and chelerythrine were 5.9 and 6.5 min, respectively. The proposed method was validated by evaluation of specificity, accuracy, precision, LOD, and LOQ. Using this method, the limit of quantification of sanguinarine and chelerythrine in tissues was estimated to be 0.5 ng/g. The average accuracies of intra- and inter-day assay fell within 90.7–105.0% and 101.6–114.0%, respectively. The mean intra- and inter-day precisions were 14.6 and 9.3% (RSD <15%), respectively. This method was suitable for accurately quantitatively determining the tissue distribution of sanguinarine and chelerythrine.

#### HPLC Method

DHCHE was quantified using a Shimadzu LC- 2010AHT system (Shimadzu Co., Kyoto, Japan), which was equipped with a LC-10ADvp pump, a SIL-10ADvp autosampler, and a SOD-10ADvp detector. Chelerythrine and DHCHE were separated on a Phenomenex Luna 5u C18(2) 100 A (4.6 × 250 mm I.D.; particle 5 μm; Phenomenex Co. USA). The mobile phase consisted of a 30/70 (v/v) mixture of solvent A (0.2% aqueous acetic acid solution) and solvent B (acetonitrile). The injection dose was 10 μl and the flow rate was 1 mL/min. The column temperature was set to 35°C and the detection wavelength was set to 285 nm. The DHCHE standard curve varied linearly in the concentration range from 0.050 to 10 μg/mL. The recovery, inter- and intra-assay coefficients of variation of DHCHE was 96.8 ± 5.2, 7.4 and 3.9% at 0.1 μg/mL, respectively. The lower limit of quantification was 0.050 μg/mL.

### Assay for NAD(P)H Quinone Oxidoreductase (NQO1) Activity

The reaction mixture contained 50 μg/mL rat liver cytosol, 0.2 mM NADH, 50 mM Tris-HCl (pH 7.4) and 40 μM 2,6-dichloro- phenolindophenol (DCPIP) in a final volume of 210 μL. The sample was transferred into the wells of the microplate with a pipette, and was measured with or without dicoumarol. Any change in absorbance was monitored at 620 nm, and NQO1 activity was calculated by using the extinction coefficient for DCPIP (ε = 21 mM^−1^cm^−1^) and expressed as nanomles of DCPIP reduced per minute per milligram of protein.

#### *In vitro* Inhibition Experiments

The inhibitory effects of sanguinarine and chelerythrine were determined by the incubation of the reaction mixture with or without substrate. The incubation mixtures for inhibition studies contained 40 μM DCPIP, 50 μg/mL rat liver cytosol and 0.2 mM NADH and different concentrations of sanguinarine or chelerythrine (0–40 μM) to determine the IC_50_ values. The K_i_ values (inhibition constant) were different concentrations of DCPIP (5–40 μM) with sanguinarine. All of the incubations and sample preparations were carried out as described for the NQO1 assay above.

#### Statistical Analysis

All results were tested by one-way analysis of variance and Dunnett's multiple comparison tests with Graphpad Prism6.0. Differences between means were determined at the *p* < 0.05 or *p* < 0.01 levels for all analyses and indicated with ^*^ and ^**^, respectively. Data were represented as mean ± S.D. of at least three replicate experiments.

## Results

### Structure Elucidation of Chelerythrine Metabolites in Rat Liver S9

**Figure 1:**
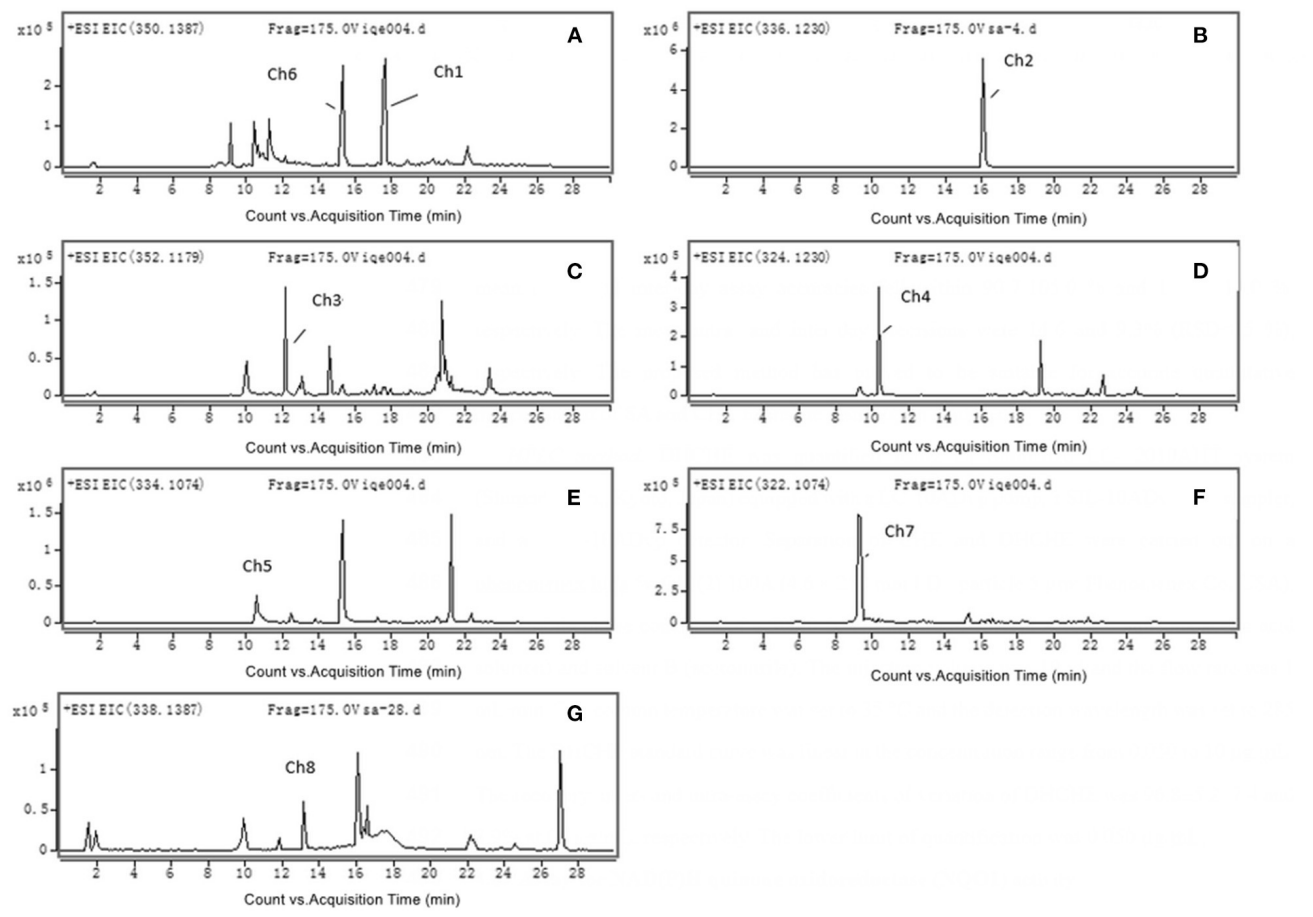
Accurate MS mass spectra chelerythrine metabolites in rat liver S9: **(A)** Ch1 (*m/z* 350); **(B)** Ch2 (*m/z* 336) and Ch6 (*m/z* 350); **(C)** Ch3 (*m/z* 352); **(D)** Ch4 (*m/z* 324); **(E)** Ch5 (*m/z* 334); **(F)** Ch7 (*m/z* 322); **(G)** Ch8 (*m/z* 338).

The accurate extracted ion chromatograms (EICs) of the chelerythrine metabolites in rat liver S9 are shown in Figures 1A–G. Eight chelerythrine metabolites (Ch1–Ch8) were detected in rat liver S9.

**Figure 2:**
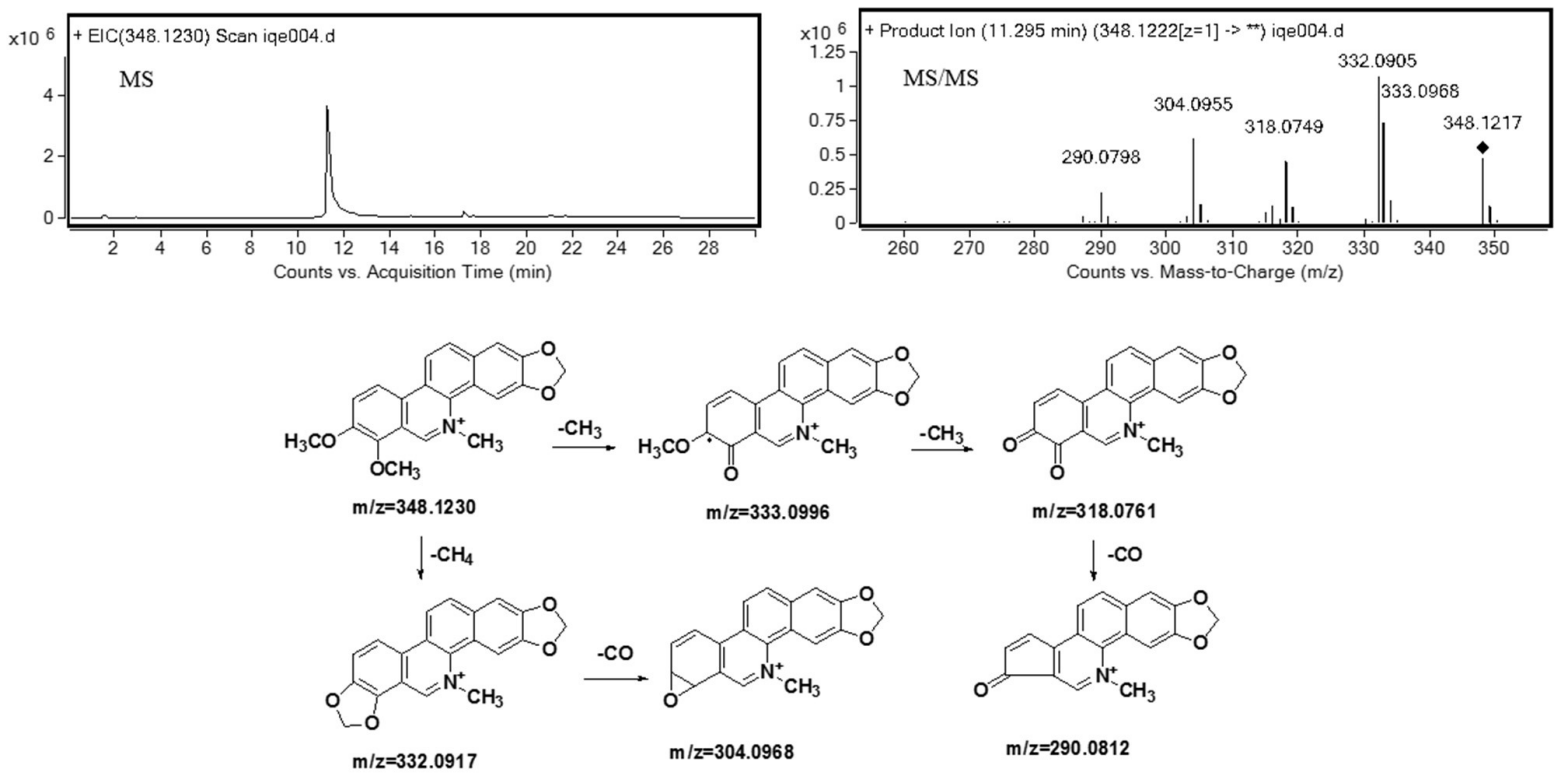
Accurate MS, MS^2^ spectra, and proposed fragmentation pathways of chelerythrine.

Understanding the fragmentation pattern of the drug substance is the first step in elucidating structures of chelerythrine metabolites. The fragmentation pattern of chelerythrine was analyzed by mass spectrometry, which provided a reference for structural identification of metabolites (shown in Table 1). It is easy to propose its product ion structures based on the structure of the parent drug and the predicated elemental compositions. As seen in Figure 2, the MS^2^ spectra of chelerythrine produces product ions at *m/z* 333 and 332, which were formed through the loss of CH_3_ and CH_4_ from the quaternary ammonium ion M^+^, respectively. The ion at *m/z* 304 was formed due to the loss of CO from *m/z* 332. The ion at *m/z* 318 lost CH_3_ from the *m/z* 333. With the further loss of CO, the ion formed *m/z* 290.

**Table 1:** The retention times (Rt), elemental compositions, observed masses and predicated masses, mass errors, and product ions of chelerythrine and its metabolites *in vitro* and *in vivo* in rats.

**Compound**	**Rt (min)**	**Elemental composition**	**Observed mass**	**Predicated mass**	**Error (ppm)**	**Product ions (product ion error, ppm)**
Chelerythrine	11.3	C_21_H_18_NO${}_{4}^{+}$ ([M]^+^)	348.1222	348.1230	−2.4	333.0968 (−8.31), 332.0905 (−3.72), 318.0749 (−3.72), 304.0955 (−4.34), 290.0798 (−4.42)
Ch1	17.6	C_21_H_20_NO${}_{4}^{+}$ ([M+H]^+^)	350.1378	350.1387	−2.53	335.1118 (−10.2), 334.1061 (−3.86), 318.1088 (−11.57), 304.0968 (−0.07)
Ch2	16.0	C_20_H_18_NO${}_{4}^{+}$ ([M+H]^+^)	336.1231	336.1230	0.2	321.0969 (−8.31), 320.0908 (−2.93), 305.0723 (13.11), 304.0948 (−6.66), 276.0987 (−11.65)
Ch3	12.2	C_20_H_18_NO${}_{5}^{+}$ ([M+H]^+^)	352.1171	352.1179	−2.42	337.0915 (−8.85), 336.0844 (−6.71), 321.0663 (−9.77), 320.0903 (−4.5), 292.0941 (−9.34)
Ch4	10.4	C_19_H_18_NO${}_{4}^{+}$ ([M+H]^+^)	324.1237	324.1230	−2.06	309.0974 (−7.01), 308.0911 (−2.07), 293.0748 (20.47), 264.0995 (−9.14)
Ch5	11.1	C_20_H_16_NO${}_{4}^{+}$ ([M]^+^)	334.1056	334.1074	−5.34	319.0827 (−3.79), 304.0632 (9.1), 291.0899 (−14.92), 276.0634 (−7.68)
Ch6	15.3	C_20_H_16_NO_5_ ([M]^+^)	350.1024	350.1023	−0.29	335.0778 (−3.06), 320.0552 (−3.06), 307.0822 (−5.77), 292.0599 (−1.83)
Ch7	9.4	C_19_H_16_NO${}_{4}^{+}$ ([M]^+^)	322.1067	322.1074	−2.12	307.0836 (−1.01), 292.0595 (−3.2), 279.0880 (−3.56), 264.0645 (−3.86)
Ch8	13.2	C_20_H_20_NO${}_{4}^{+}$ ([M+H]^+^)	338.1369	338.1387	−5.28	323.1104 (−16.72), 322.1035 (−13.66), 306.1024 (−34.62)292.0950 (−6.23), 278.0793 (−6.72)
Ch9	11.4	C_21_H_18_NO${}_{5}^{+}$ ([M]^+^)	364.1178	364.1179	−0.41	349.0928 (−4.8), 348.0869 (0.72), 334.0722 (3.6), 320.0920 (0.83), 306.0773 (3.97)
Ch10	14.9	C_21_H_20_NO${}_{5}^{+}$ ([M+H]^+^)	366.1339	366.1336	0.82	351.1081 (−5.78), 350.1008 (−5.78), 334.1026 (−14.37), 320.0910 (−2.3), 306.0763 (0.71)
Ch11	10.8	C_20_H_16_NO${}_{5}^{+}$ ([M]^+^)	350.1007	350.1023	−4.57	335.0781 (−2.16), 320.0542 (−3.59), 307.0824 (−4.92), 292.0598 (−2.72)
Ch12	11.4	C_20_H_16_NO${}_{4}^{+}$ ([M]^+^)	334.1076	334.1074	0.65	319.0840 (0.28), 304.0610 (1.86), 291.0890 (0.02), 276.0668 (4.64)

The retention time of metabolite Ch1 was 18.4 min, and it showed the [M+H]^+^ ion at *m/z* 350, which was 2 Da higher than chelerythrine. The MS^2^ spectrum of Ch1 (see Table 1) produced ions at *m/z* 335 and 334, which were formed due to the loss of CH_3_ and CH_4_ from *m/z* 350, respectively. The ion at *m/z* 318 was produced by the loss of CH_3_OH from *m/z* 350. The ion at *m/z* 304 was formed through the loss of CH_2_O from *m/z* 334. The retention time of Ch1 was the same, [M+H]^+^ ion and MS^2^ spectrum as authentic DHCHE. As a result, Ch1 was identified as DHCHE.

The retention time of metabolite Ch2 was 16.0 min, and it showed the [M+H]^+^ ion at *m/z* 336, which was 12 Da lower than chelerythrine, thereby indicating that it could be chelerythrine demethylenation metabolite or DHCHE O-demethylation metabolite. The MS^2^ spectrum of Ch2 (see Table 1) produced ions at *m/z* 321 and 320, which were formed through the loss of CH_3_ and CH_4_ from *m/z* 336, respectively. The ion at *m/z* 305 may be formed due to the loss of CH_3_ from *m/z* 320. The ion at *m/z* 304 was produced by the loss of CH_3_OH from *m/z* 336. The ion at *m/z* 304 saw further loss of CO, forming *m/z* 276. Moreover, the MS^2^ spectrum of Ch2 showed product ions at *m/z* 321, 320, 304 (see Table 1), which were 14 Da lower than the product ions at *m/z* 335, 334, 318 of DHCHE. Therefore, metabolite Ch2 could be DHCHE O-demethylation metabolite located at the positions on the C7 or C8 bond.

The retention time of metabolite Ch3 was 12.2 min, and it showed the [M+H]^+^ ion at *m/z* 352, which was 16 Da higher than Ch2. The MS^2^spectrum of Ch3 (see Table 1) produced ions at *m/z* 337, 336, 321, 320, 292, which were 16 Da higher than the product ions at *m/z* 321, 320, 305, 304, 276 of Ch2. The MS^2^spectrum of Ch3 produced ions at *m/z* 337 and 336, which were formed due to the loss of CH_3_ and CH_4_ from *m/z* 352, respectively. The ion at *m/z* 321 was formed through the loss of CH_3_ from *m/z* 336. The ion at *m/z* 320 was produced by the loss of CH_3_OH from *m/z* 352. The ion at *m/z* 320 saw a further loss of CO, forming *m/z* 292. Based on these results, we preliminarily identified that Ch3 was a hydroxylation metabolite of Ch2 located at the positions on the phenyl ring.

The retention time of metabolite Ch4 was 10.4 min, and it showed the [M+H]^+^ ion at *m/z* 324, which was 12 Da lower than Ch2, thus suggesting that it may be Ch2 demethylenation metabolite. The MS^2^ spectrum of Ch4 (see Table 1) produced ions at *m/z* 309 and 308, which were formed due to loss of CH_3_ and CH_4_ from *m/z* 324, respectively. The ion at *m/z* 293 may be formed through the loss of CH_3_ from *m/z* 308. The ion at *m/z* 264 was produced by the loss of CHO from *m/z* 308. Moreover, the MS^2^ spectrum of Ch4 showed product ions at *m/z* 309, 308, 293, which were 12 Da lower than the product ions at *m/z* 321, 320, 305 of Ch2. Therefore, metabolite Ch4 could be Ch2 O-demethylenation metabolite at the methylenedioxy group.

The retention time of metabolite Ch5 was 11.1 min, and it showed the [M]^+^ ion at *m/z* 334, which was 14 Da lower than chelerythrine. We did not detect the [M-16]^+^ ion, so the product ions could not be N-demethylenatedchelerythrine metabolite, but were probably O-demethylenatedchelerythrine metabolite. The MS^2^ spectrum of Ch5 (see Table 1) produced ions at *m/z* 319, which were formed due to the loss of CH_3_ from *m/z* 334. The ion at *m/z* 304 was formed through the loss of CH_3_ from *m/z* 319. The ion at *m/z* 319 saw further loss of CO, forming *m/z* 291. The ion at *m/z* 276 was produced by the loss of CH_3_ from *m/z* 304. Consequently, metabolite Ch5 was identified as O-demethylated chelerythrine metabolite located at the positions on the C7 or C8 bond.

The retention time of the metabolite Ch6 was 15.3 min, and it showed the [M]^+^ ion at *m/z* 350, which was 16 Da higher than Ch5. Ch6 might be a hydroxylated derivative of Ch5. The MS^2^spectrum of Ch6 (see Table 1) produced ions at *m/z* 335, which were formed due to the loss of CH_3_ from *m/z* 350. The ion at *m/z* 335 saw further loss of CO, forming *m/z* 307. The ion at *m/z* 320 was formed though the loss of CH_3_ from *m/z* 335. The ion at *m/z* 320 saw further loss of CO, forming *m/z* 292. Moreover, the ions of Ch6 showed a shift of +16 Da from those of Ch5. As a result, the possible hydroxyl could be located on a phenyl ring. Based on these results, the structure of Ch6 was tentatively identified a hydroxylation metabolite of Ch5 located at the positions on the phenyl ring.

The retention time of metabolite Ch7 was 9.4 min, and it showed the [M]^+^ ion at *m/z* 322, which was 12 Da lower than those from Ch5, corresponding to possible O-demethylenated Ch5 metabolite. The MS^2^spectrum of Ch7 produced ion at *m/z* 307, which were formed due to the loss of CH_3_ from *m/z* 322. The ion at *m/z* 307 saw further loss of CH_3_, forming *m/z* 292. The ion at *m/z* 279 was formed though the loss of COfrom *m/z* 307. The ion at *m/z* 264 was produced by the loss of CO from *m/z* 292. Therefore, we preliminarily identified that metabolites Ch7 was the ring-cleavage of Ch5, and then O-demethylenation at the methylenedioxy group.

The retention time of metabolite Ch8 was 13.2 min, and it showed the [M+H]^+^ ion at *m/z* 338, which was 12 Da lower than DHCHE, thus suggesting that it may be DHCHE demethylenation metabolite. The MS^2^ spectrum of Ch8 (see Table 1) produced ions at *m/z* 323 and 322, which were formed due to loss of CH_3_ and CH_4_ from *m/z* 338, respectively. The ion at *m/z* 306 was formed though the loss of CH_3_OH from *m/z* 338. The ion at *m/z* 306 saw further loss of CO, forming *m/z* 278. The ion at *m/z* 292 was produced by the loss of CH_2_O from *m/z* 322. Therefore, metabolite Ch8 could be DHCHE O-demethylenation metabolite at the methylenedioxy group.

### Metabolite Profiles of Chelerythrine in Plasma, Feces, and Urine in Rats After Intragastric Administration of Chelerythrine

The chelerythrine metabolites detected in plasma, feces, and urine of rats are shown in Table 2. Three hours after intragastric administration of chelerythrine, no parent drug and metabolites were detected in the plasma of the male and female rats. Only the metabolites Ch2 and Ch5 were found in the urine of 0–12 h male rats. Eight and seven metabolites were observed in the feces of female and male rats during 0–12 h after intragastric administration of chelerythrine, respectively. They were Ch1, Ch2, Ch5, Ch8, Ch9, Ch10, Ch11, and Ch12, and seven of these metabolites were found in male rat feces of 0–12 h except for Ch10. Supplementary Figure 1 shows the accurate EICs of female rat feces after a single dose oforal administration of chelerythrine for 0–12 h (See Supplemental Material). Except for chelerythrine, only Ch1 was observed in the feces of female rats from 12 to 24 h after intragastric administration of chelerythrine.

**Table 2:** Summary of chelerythrine metabolites detected in the plasma, urine, feces, and tissues of rats.

**Compound**	**Female**					**Male**				
	**Plasma**	**Urine**	**Feces**	**Plasma**	**Urine**	**Feces**	**Heart**	**Liver**	**Spleen**	**Lung**	**Kidney**									
	**3 h**	**0–12 h**	**12–24 h**	**0–12 h**	**12–24 h**	**3 h**	**0–12 h**	**12–24 h**	**0–12h**	**12–24 h**	**24 h**	**48 h**	**24 h**	**48 h**	**24 h**	**48 h**	**24 h**	**48 h**	**24 h**	**48 h**
Chelerythrine	ND	ND	ND	√	√	ND	ND	ND	√	√	ND	ND	ND	ND	ND	ND	ND	ND	ND	ND
DHCHE	ND	ND	ND	√	√	ND	ND	ND	√	ND	ND	ND	ND	ND	ND	ND	ND	ND	ND	ND
Ch2	ND	ND	ND	√	ND	ND	√	ND	√	ND	ND	ND	ND	ND	ND	ND	ND	ND	ND	ND
Ch3	ND	ND	ND	ND	ND	ND	ND	ND	ND	ND	ND	ND	ND	ND	ND	ND	ND	ND	ND	ND
Ch4	ND	ND	ND	ND	ND	ND	ND	ND	ND	ND	ND	ND	ND	ND	ND	ND	ND	ND	ND	ND
Ch5	ND	ND	ND	√	ND	ND	√	ND	√	ND	ND	ND	ND	ND	ND	ND	ND	ND	ND	ND
Ch6	ND	ND	ND	ND	ND	ND	ND	ND	ND	ND	ND	ND	ND	ND	ND	ND	ND	ND	ND	ND
Ch7	ND	ND	ND	ND	ND	ND	ND	ND	ND	ND	ND	ND	ND	ND	ND	ND	ND	ND	ND	ND
Ch8	ND	ND	ND	√	ND	ND	ND	ND	√	ND	ND	ND	ND	ND	ND	ND	ND	ND	ND	ND
Ch9	ND	ND	ND	√	ND	ND	ND	ND	√	ND	ND	ND	ND	ND	ND	ND	ND	ND	ND	ND
Ch10	ND	ND	ND	√	ND	ND	ND	ND	ND	ND	ND	ND	ND	ND	ND	ND	ND	ND	ND	ND
Ch11	ND	ND	ND	√	ND	ND	ND	ND	√	ND	ND	ND	ND	ND	ND	ND	ND	ND	ND	ND
Ch12	ND	ND	ND	√	ND	ND	ND	ND	√	ND	ND	ND	ND	ND	ND	ND	ND	ND	ND	ND

*Ch followed by number, metabolite; √, detected; ND, not detected*.

### Structure Elucidation of Chelerythrine Metabolites in Rat *in vivo*

Except for four metabolites (Ch1, Ch2, Ch5, and Ch8) observed in rat liver S9, four metabolites (Ch9–Ch12) were also found *in vivo*. The retention time of Metabolite Ch9 was 11.4 min, and it showed the [M]^+^ ion at *m/z* 364, which was 16 Da higher than chelerythrine. Ch9 might be a hydroxylated derivative of chelerythrine. The MS^2^ spectra of Ch9 (see Table 1) produced ions at *m/z* 349 and 348, which were formed due to the loss of CH_3_ and CH_4_ from *m/z* 364, respectively. The ion at *m/z* 320 was formed though the loss of CO from *m/z* 348. The ion at *m/z* 334 lost CH_3_ from the *m/z* 349. With the further the loss of CO, the ion formed *m/z* 306.Therefore, metabolite Ch9 could be a hydroxylation metabolite of chelerythrine located at the positions on the phenyl ring.

The retention time of metabolite Ch10 was 14.9 min, and it showed the [M+H]^+^ ion at *m/z* 366, which was 16 Da higher than DHCHE. Ch10 might be a hydroxylated metabolite of DHCHE. The MS^2^ spectra of Ch10 (see Table 1) produced ions at *m/z* 351 and 350, which were formed due to the loss of CH_3_ and CH_4_ from *m/z* 366, respectively. The ion at *m/z* 334 was formed though the loss of CH_3_OH from *m/z* 366. With the further the loss of CO, the ion formed *m/z* 306. The ion at *m/z* 320 was produced by the loss of CH_2_O from *m/z* 350. Therefore, metabolite Ch10 could be a hydroxylation metabolite of DHCHE located at the positions on the phenyl ring.

The retention time of metabolite Ch11 was 10.8 min, and it showed the [M]^+^ ion at *m/z* 350, which was also 16 Da higher than Ch5. Ch11 might be a hydroxylated derivative of Ch5. Ch11 and Ch6 had the same MS^2^ spectrum and [M]^+^ ion. As a result, Ch11 was preliminarily identified as an isomer of Ch6. The retention time of metabolite Ch12 was 11.4 min, and it showed the [M]^+^ ion at *m/z* 334, and Ch12 and Ch5 had the same MS^2^ spectrum and [M]^+^ ion. Consequently, Ch12 was preliminarily identified as an isomer of Ch5.

### Metabolism and Tissue Distribution of Sanguinarine and Chelerythrine After Intragastric Administration of *M. cordata* Extracts

As shown in Table 2, no metabolites of sanguinarine and chelerythrine were found in 24 and 48 h rat tissues after 3 weeks of intragastric administration of *M. cordata* extracts (containing 40% sanguinarine and 20% chelerythrine). The metabolite profiles of sanguinarine in plasma, urine, and feces in rats were examined because the *M. cordata* extracts contained sanguinarine. No parent drug or metabolites were observed in rat plasma at 3 h and urine of 0–24 h after intragastric administration of *M. cordata* extracts. In addition to the parent drug, two metabolites were observed in the feces of both male and female rats during 0–24 h (See Supplementary Figure 2).

As seen in Table 3, the retention time of sanguinarine was 11.0 min, and it showed the [M]^+^ ion at *m/z* 332. The ions at *m/z* 317 and *m/z* 304 were formed due to the loss of CH_3_ and CO from *m/z* 332, respectively. The ion at *m/z* 304 further lost CH_2_O, forming *m/z* 274. And the ion at *m/z* 246 lost CO from the ion at *m/z* 274.

**Table 3:** The retention times (Rt), elemental compositions, observed masses and predicated masses, mass errors, and product ions of sanguinarine and its metabolites *in vivo* in rats.

**Compound**	**Rt (min)**	**Elemental composition**	**Observed mass**	**Predicated mass**	**Error (ppm)**	**Product ions (product ion error, ppm)**
Sanguinarine	11.0	C_20_H_14_NO${}_{4}^{+}$([M]^+^)	332.0911	332.0917	−1.91	317.0672 (−3.34), 304.0955 (−4.34), 274.0853 (−3.48), 246.0903 (−4.23)
S1	18.0	C_20_H_16_NO${}_{4}^{+}$([M+H]^+^)	334.1074	334.1074	−0.05	319.0817 (−6.95), 318.0755 (−1.84), 304.0944 (−7.98), 276.1012 (−2.56)
S2	15.3	C_20_H_14_NO${}_{5}^{+}$([M]^+^)	348.0866	348.0866	−0.14	333.0633 (0.38), 320.0905 (−3.87), 318.0736 (−7.84), 305.0671 (−3.81), 290.0781 (−10.62)
S3	13.4	C_19_H_16_NO${}_{4}^{+}$([M+H]^+^)	322.1072	322.1074	−0.57	307.0864 (8.14), 306.0759 (−0.6), 292.0948 (−6.94), 264.1008 (−4.2)

The retention time of Metabolite S1 was 18.0 min, and it showed the [M+H]^+^ ion at *m/z* 334 (see Table 3), which was 2 Da higher than sanguinarine. The ions at *m/z* 319 and *m/z* 318 were formed due to loss of CH_3_ and CH_4_ from *m/z* 334, respectively. The ion at *m/z* 334 further lost CH_2_O, forming *m/z* 304. The ion at *m/z* 304 also lost CO, forming the ion at *m/z* 276. S1 possessed the same retention time, product ions as authentic DHSA. Therefore, S1 was identified as DHSA.

The retention time of metabolite S2 was 15.3 min, and it showed the [M]^+^ ion at *m/z* 348 (see Table 3), which indicated it may be an oxidative metabolite of sanguinarine. The ion at *m/z* 348 lost CH_3_, forming the ion at *m/z* 333. It showed that the position of oxidative could not be located on CH_3_ of N5. The ion at *m/z* 320 and *m/z* 318 were formed due to loss of CO and CH_2_O from the ion at *m/z* 348, respectively. The ion at *m/z* 320 further lost CH_3_, forming *m/z* 305. The ion at *m/z* 318 also lost CO forming *m/z* 290. Therefore, metabolite S2 could be an oxidative metabolite of sanguinarine located at the positions on the phenyl ring.

The retention time of metabolite S3 was 13.4 min (see Table 3), and it showed the [M+H]^+^ ion at *m/z* 322, which was 12 Da lower than DHSA, corresponding to the demethylenation metabolite. The product ion at *m/z* 307 and *m/z* 306 was formed due to loss of CH_3_ and CH_4_ from the ion at *m/z* 322, respectively. The ion at *m/z* 322 lost CH_2_O, forming the ion at *m/z* 292. The ion further saw the loss of CO, forming *m/z* 264. Therefore, metabolite S3 could be DHSA O-demethylenation metabolite at the methylenedioxy group.

The HPLC-QqQ MS method was used for further analysis of the rat tissues and for the qualitative determination of sanguinarine and chelerythrine. As shown in Table 4, the distribution of sanguinarine and chelerythrine in rat cecal contents and tissue after intragastric administration of *M. cordata* extracts for 3 weeks. The concentrations of chelerythrine in the liver of 48 h after dosing and spleen of 24 h after dosing were 20.64 and 24.04 ng/g, respectively. The concentrations of sanguinarine and chelerythrine in other tissues were <11 ng/g.

**Table 4:** The residue result of sanguinarine and chelerythrine in rat tissues (ng/g).

**Tissues**	**Sanguinarine**		**Chelerythrine**	
	**24 h**	**48 h**	**24 h**	**48 h**
Liver	10.38	9.8	9.39	20.64
Heart	2.91	2.57	2.79	3.87
Spleen	2.01	1.91	24.04	1.1
Lung	2.29	2.01	1.21	1.71
Kidney	3.77	5.65	1.62	0.91

### Effects of Inhibitors on Reduction of Chelerythrine Iminium Bond in Rat Liver Cytosol

**Figure 3:**
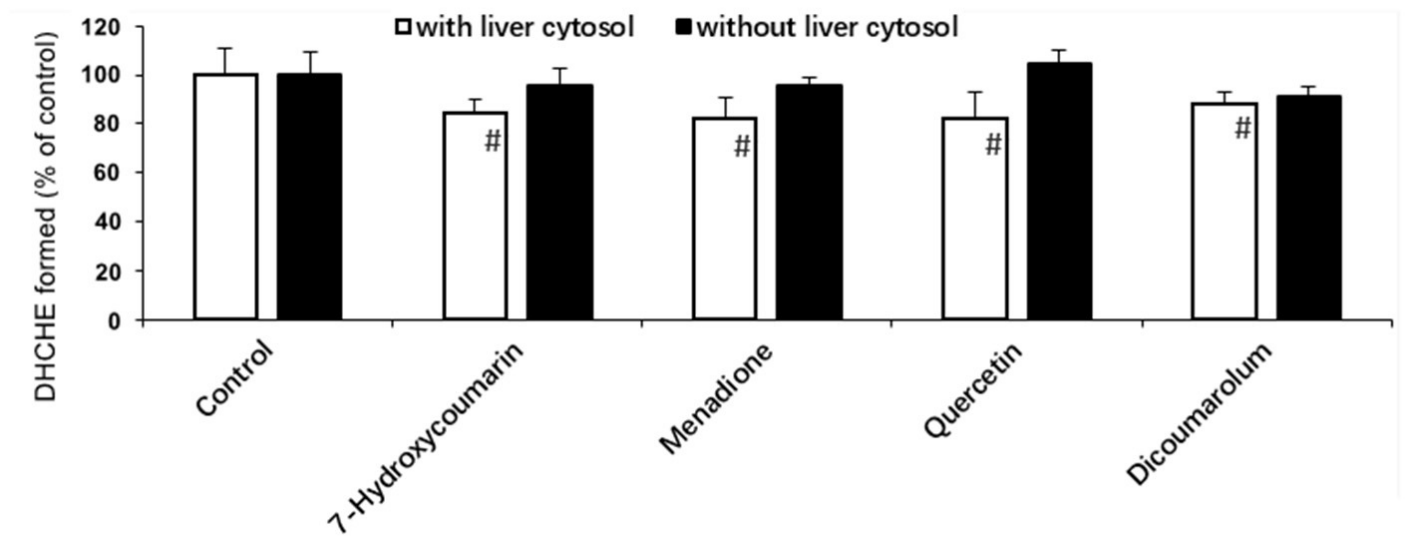
Effect of some inhibitors on the chelerythrine iminium bond reduction to DHCHE by rat liver cytosol. #*p* < 0.05, significant when compared to control without inhibitor.

Figure 3 showed that all selected inhibitors did not exhibit obvious effects on the DHCHE formation only in the presence of NADPH. However, it was demonstrated that the addition of quercetin, 7-hydroxycoumarin, menadione, and dicoumarol could inhibit the iminium bond reduction of chelerythrine in rat liver cytosol in the presence of NADPH.

### Effect on N QO1 Activity *in vivo* and *in vitro*

#### Effect on NQO1 Activity *in vivo*

**Figure 4:**
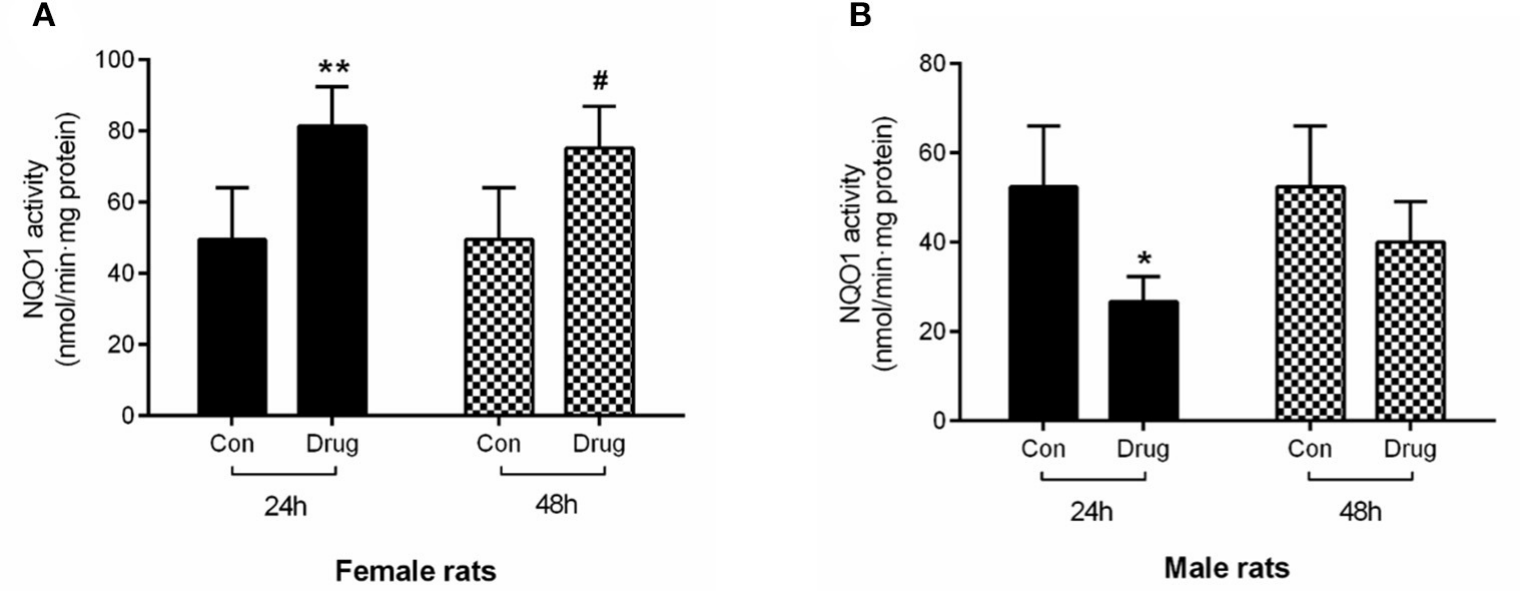
Assessment of *in vivo* NQO1 activity in female rats **(A)** and male rats **(B)** following intragastric administered with *M. cordata* extracts. Con, Control group; Drug, administrated with *M. cordata* extracts. *^/#^*P* < 0.05, **^/*##*^*P* < 0.01 vs. respective control group of female and male rat.

The activity of the NQO1 enzyme in the female rats treated with *M. cordata* extracts increased by 64.50 and 52.88% at 24 and 48 h, respectively, in comparison with control rats (Figure 4A). On the contrary, the activity of the NQO1 enzyme in male rats decreased by 47.55 and 23.47% at 24 and 48 h, which was statistically significance in contrast to the control (Figure 4B).

#### The Effect of Sanguinarine and Chelerythrine on NQO1 Activity *in vitro*

The effects of chelerythrine and sanguinarine on NQO1 activity are demonstrated in Table 5. The results showed that chelerythrine had a weak impact on NQO1 activity compared to dicoumarol with strong effect on that, and sanguinarine had a statistically significant decline compared to the control, and the IC_50_ value was 111.57 μM.

**Table 5:** Effect of compounds on rat cytosolic NAD(P)H: quinone oxidoreductase activities.

**Compounds**	**Concentrtion (μM)**	**Rats**	
		**NQO1 activities**	**IC_**50**_ (μM)**
Control	–	67.14 ± 4.82	–
Dicoumarol	40.00	6.18 ± 0.67^**^	0.008 ± 0.002
Chelerythrine	40.00	64.90 ± 4.24	–
Sanguinarine	40.00	45.08 ± 4.34^**^	111.57 ± 6.17

*NAD(P)H, quinone oxidoreductase activity is presented as nanomolars of 2,6-dichlorophenolindophenol reduced per minute per milligram of protein.*

** *P < 0.01.*

## Discussion

This was the first study that detailed structural elucidations of chelerythrine metabolites in rats *in vitro and vivo* using an accurate and rapid HPLC/QqTOF-MS method. A total of twelve metabolites of chelerythrine were observed *in vitro* and *in vivo*. Among of these metabolites, Ch8 was identified for the first time. Eight metabolites of chelerythrine were found in rat liver S9. In addition to four metabolites (Ch1, Ch2, Ch5, Ch8) observed in rat liver S9, four other metabolites (Ch9–Ch12) were also found in rat feces. Metabolite Ch1 (DHCHE) and Ch2 were the main metabolites in rats *in vivo* and *in vitro*, respectively. And Ch2 was formed by the O-demethylation from Ch1. So, we speculated O-demethylation of DHCHE has an advantage *in vitro* over *in vivo*. The difference on the metabolite profiles of chelerythrine between *in vitro* and *in vivo* was observed.

**Figure 5:**
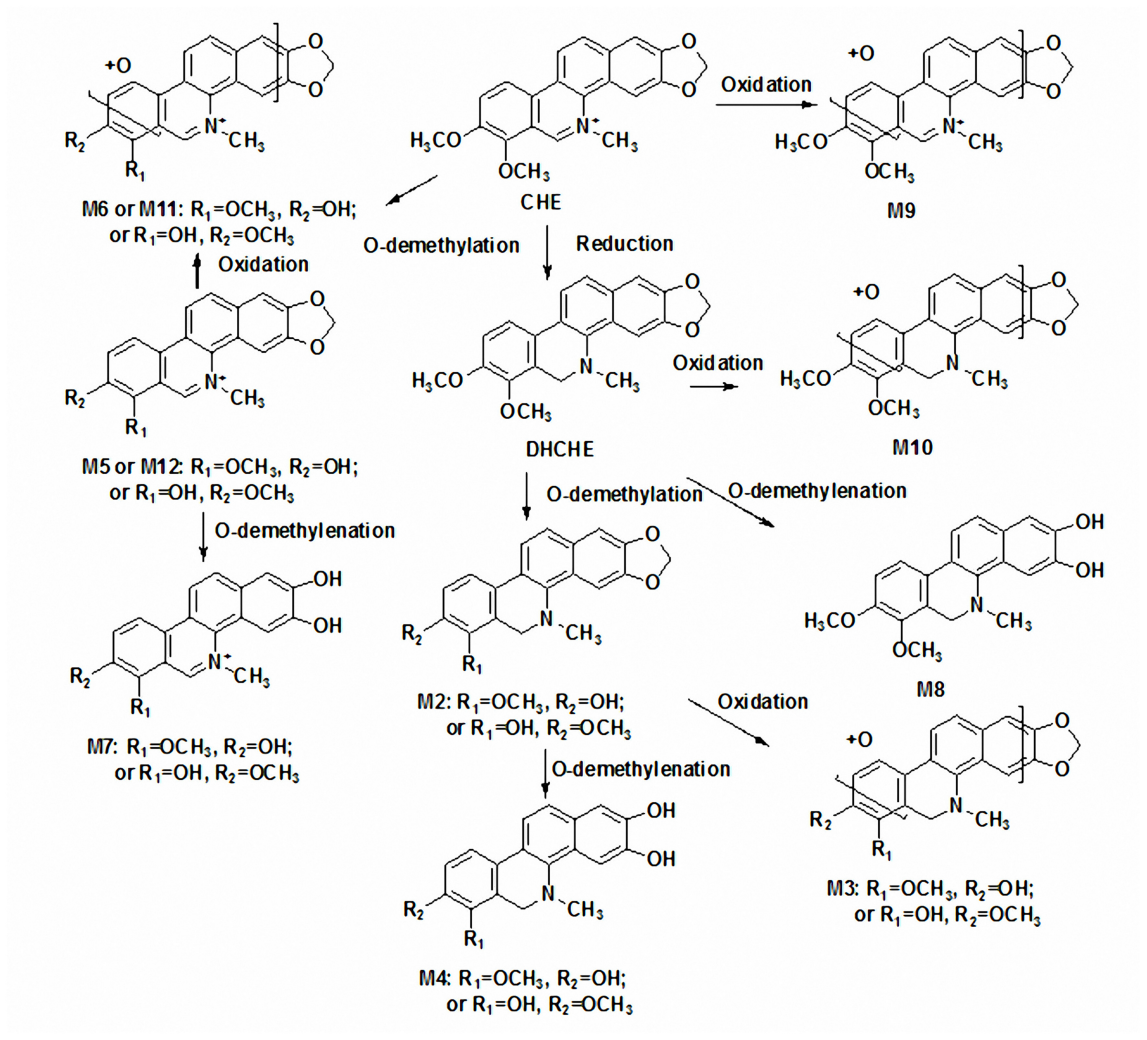
The possible metabolic pathways of chelerythrine in liver S9 and *in vivo*.

According to these results, the possible metabolic pathways of chelerythrine in liver S9 were proposed (Figure 5). The present results indicated that the reduction of the iminium bond of chelerythrine and subsequent O-demethylation were the common metabolic pathway of chelerythrine in rat *in vitro and vivo*. But the reduction of the iminium bond of chelerythrine and subsequent O-demethylation was the main metabolic pathway of chelerythrine in rat liver S9 while the reduction of the iminium bond of chelerythrine was the main metabolic pathway of chelerythrine in rat *in vivo*. The O-demethylenated metabolites of DHCHE, O-demethylated and subsequent oxidated metabolites of chelerythrine were also the common metabolic pathways of chelerythrine in rat *in vitro and vivo*. Additionally, the O-demethylation and subsequent O-demethylenation of chelerythrine, DHCHE, can be observed in rat liver S9, and the O-demethylated and subsequent oxidated O-demethylenated of DHCHE were found in rat liver S9. The oxidated metabolite of chelerythrine and DHCHE can be detected in rat *in vivo*.

No parent drug or metabolite**s** were observed in the plasma of female and male rat at 3 h following intragastric administration of chelerythrine. Pharmacokinetic study indicated that the C_max_, T_max_ and T_1/2_ of chelerythrine were 5.06 ng/ml, 1.67, 2.82 h, respectively. No parent drug or metabolite**s** were found in urine of female rats during 0–12 h after intragastric administration of chelerythrine. But two metabolites (Ch2 and Ch5) identified in liver S9 were observed in the urine of male rats during 0–12 h following intragastric administration of chelerythrine, and Ch2 and Ch5 were not detected in the urine of male rats during the 12–24 h following intragastric administration of chelerythrine. The result indicated that the absorption and excretion of chelerythrine was fast in rats. Most metabolites were observed in rat feces. Eight and seven chelerythrine metabolites were found in the feces of female and male rats, respectively. The results were consistent with the previously published results (6, 20), a majority of the chelerythrine and metabolites were excreted in the feces in 12 h during the 0–12 h after drug withdrawal and a small part of the alkaloid was absorbed.

A total of three sanguinarine metabolites were observed following the intragastric administration of 5 mg/kg (containing about 2 mg/kg dose of sanguinarine and 1 mg/kg dose of chelerythrine) *M. cordata* extracts. The main metabolite DHSA was observed in this experiment, consistent with previous reports (13). Except S1, S2, and S3, other reported metabolites were not identified in rats *in vivo*. We inferred that the reason for this may be related to the dosage and sensitivity of the HPLC/QqTOF-MS method.

In China, the extracts of *M. cordata* containing chelerythrine and sanguinarine were approved as the first Traditional Chinese Veterinary Medicine in feed. Products containing *M. cordata* extract, with a bitter flavor, have been used as a naturally-derived appetizing agent for addition to the feed of production animals including bovine (21, 22), poultry (23, 24), fish (25), and swine (26, 27). With the increasing attention on food safety, it was important to study the residue of sanguinarine and chelerythrine in tissues following intragastric administration with *M. cordata* extracts for 3 weeks. After 3 weeks of intragastric administration with *M. cordata* extracts by using the HPLC/QqTOF-MS method, no sanguinarine and chelerythrine or their metabolites were found in the spleen, lung, kidney, liver or heart. Further, a HPLC-MS/MS was used for imploring the distribution in tissues of sanguinarine and chelerythrine. The concentrations of chelerythrine in the liver 48 h after dosing and spleen of 24 h after dosing were 20.64 and 44.04 ng/ml, which were higher than that in others tissues (< 15 ng/g). The concentrations of sanguinarine in the tissues were also < 15 ng/g. The results indicated that sanguinarine and chelerythrine are distributed in different tissues, keeping consistent with previous research (6). Therefore, the active component of photogenic feed additives for livestock may exist in certain tissue residues after feeding.

**Figure 6:**
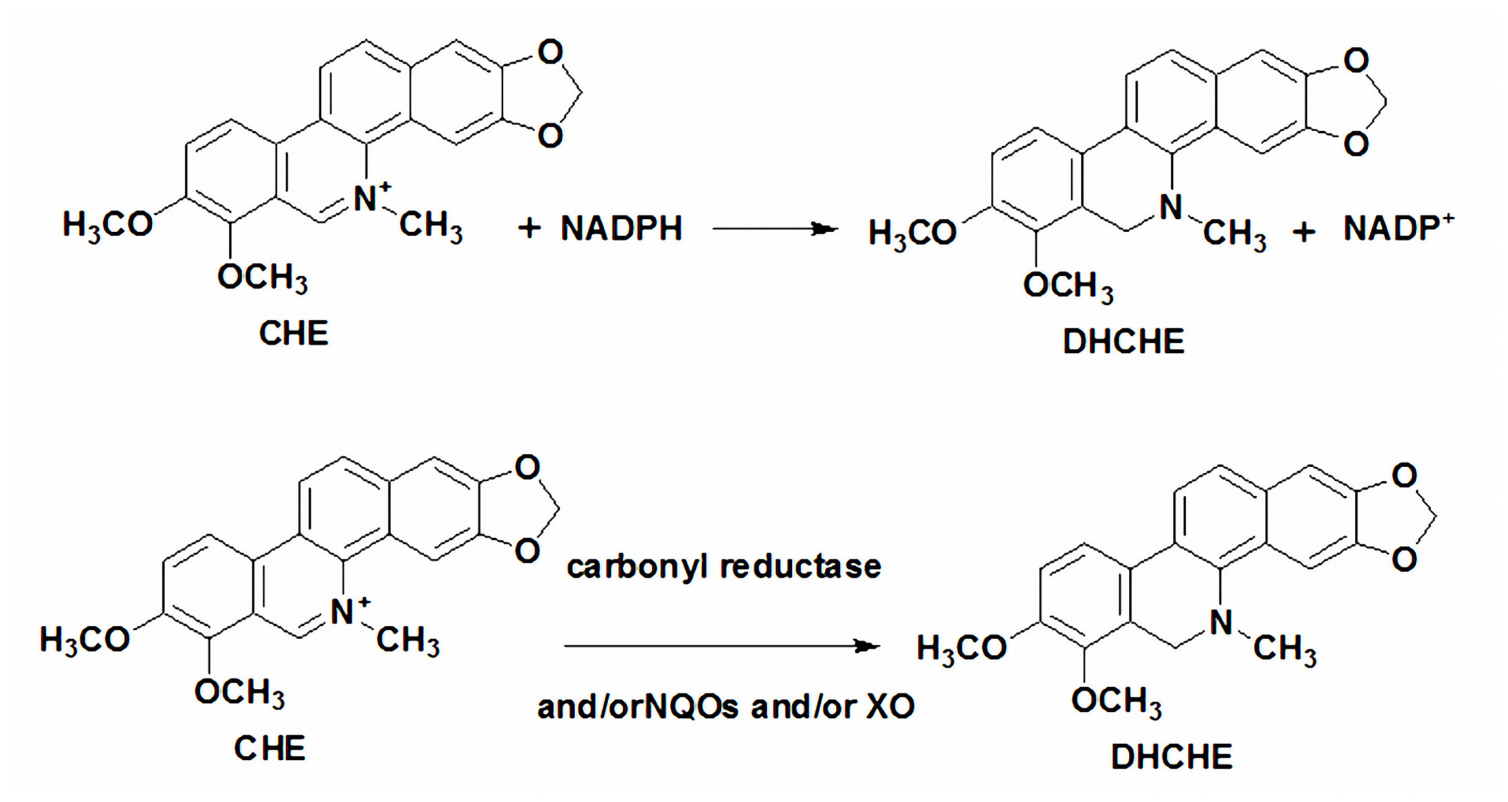
The mechanism of chelerythrine reduction in the rat liver cytosol.

The chelerythrine transformed into DHCHE by reducing the iminium bond. This may be an important detoxication pathway of chelerythrine in humans and in animals. Previous reports showed that the reduction of sanguinarine iminium bond in cytosolic may lead to carbonyl reductase and/or NQOs (13). But the mechanism of chelerythrine reduction in animals is not clear. In the present study, the inhibitor effects of iminium bond reduction of chelerythrine in rat liver cytosol were investigated. Xanthine oxidase inhibitor 7-hydroxycoumarin, NQO1 inhibitor dicoumarol, NQO2 and carbonyl reductase inhibitor quercetin and menadione-a specific substrate of carbonyl reductase and NQO2 had significant effect on DHCHE formation in rat liver cytosol in the presence of NADPH. The results that indicated XO, NQO1, NQO2, and carbonyl reductase are involved in chelerythrine reduction. We also propose the mechanism of chelerythrine reduction in the rat liver cytosol as shown in Figure 6. The difference of reduction metabolism mechanism between sanguinarine and chelerythrine may be related to their structure.

Sanguinarine and chelerythrine are active substances in *M. cordata* extracts. But the effects of sanguinarine and chelerythrine on drug-metabolizing enzymes *in vivo* have not been reported. In this study, the impact of *M. cordata* extracts on the activity of NQO1 in male and female rats were investigated. The results indicated that the treatment of rats with *M. cordata* extracts induced the activity of NQO1 in female rats, but inhibition activity in male rats. Clinical and experimental observations show that the female's response to dietary sodium chloride (salt sensitivity) may be different from that of the male due to the intermediate effects of the sex hormone patterns and gender-related genetic factors (19). Therefore, we suspected that the gender difference in drug-metabolizing enzymes between sanguinarine and chelerythrine might be related to the difference in enzyme activities that were regulated by sex hormones. Further, we investigated the impact of sanguinarine and chelerythrine on NQO1 activity. The result indicated chelerythrine had a weak impact on NQO1 activity, but sanguinarine inhibited NQO1 activity. It provides more comprehensive information in the chelerythrine metabolism and mechanism, and will be helpful to application of *M. cordata* extracts in veterinary and human medicine.

## Conclusions

The current study investigated comprehensively the biotransformation of chelerythrine in the urine, feces, and plasma of rats following intragastric administration of chelerythrine and *in vitro* in rat. The tissue distribution of sanguinarine and chelerythrine was explored further after intragastric administration of *M. cordata* extracts for 3 weeks. The effects of cytosolic reductase inhibitors on chelerythrine reduction and the impact of chelerythrine and sanguinarine on the activity of NQO1 *in vitro and in vivo* were also examined in this study. These findings provide a reference for the clinical veterinary application and food safety evaluation of *M. cordata* extracts.

## Data Availability Statement

The original contributions presented in the study are included in the article/Supplementary Material, further inquiries can be directed to the corresponding author/s.

## Ethics Statement

The animal study was reviewed and approved by the Institutional animal care and use committee of Hunan Research Center.

## Author Contributions

Z-YL and Y-JH participated in creating the experimental design. Y-JH and Z-YZ performed the handling of animals and sample collection. C-YH, Y-JH, and Y-SL analyzed the data and discussed the obtained results. C-YH, Y-JH, and Z-YL wrote manuscript. All authors contributed to the article and approved the submitted version.

## Conflict of Interest

Y-JH was employed by company Hunan Prima Drug Research Center. The remaining authors declare that the research was conducted in the absence of any commercial or financial relationships that could be construed as a potential conflict of interest.
