# Supplementary material for: Metabolism and Tissue Distribution of Chelerythrine and Effects of Macleaya Cordata Extracts on Liver NAD(P)H Quinone Oxidoreductase
Source: Front Vet Sci. 2021 May 26;8:659771. doi: 10.3389/fvets.2021.659771 (PMC8187775; doi:10.3389/fvets.2021.659771)
Supplement: Supplementary file 1 [file Data_Sheet_1.doc]

Supplementary Figure


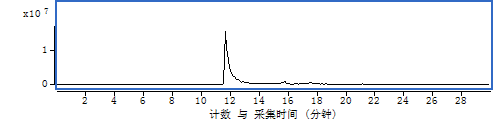


+ EIC（348.1230）

（A）

CHE


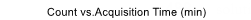


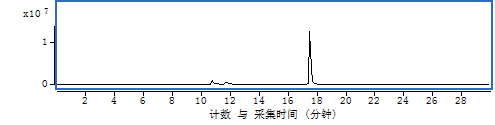


+ EIC（350.1387）

（B）

Ch1


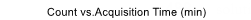


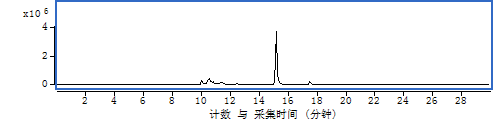


+ EIC（336.1230）

Ch2

（C）


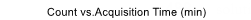


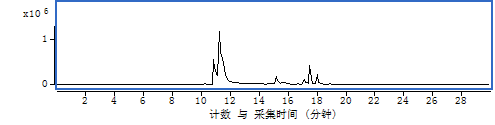


Ch12

+ EIC（334.1074）

（D）

Ch5

#
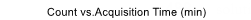


#
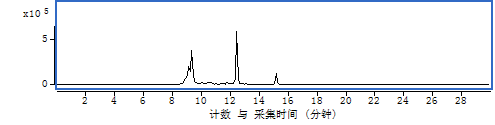


+ EIC（338.1387）

Ch8

（E）


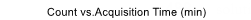


#
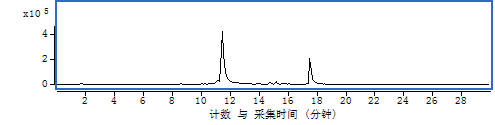


（F）

Ch9

+ EIC（364.1179）

#
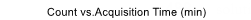

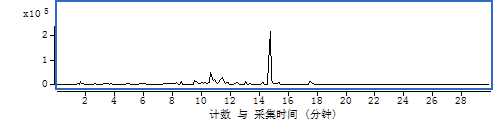


+ EIC（366.1336）

（G）

Ch10


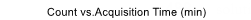


#
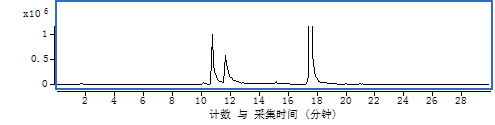


+ EIC（350.1023）

（H）

Ch11


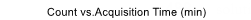


**Supplementary Figure S-1.** The accurate EIC of CHE metabolites in female SD rat feces at 0-12 h: (A) CHE (*m/z* 348); (B) Ch1 (*m/z* 350); (C) Ch2 (*m/z* 336); (D) Ch3 and Ch8 (*m/z* 334); (E) Ch4 ( *m/z* 338); (F) Ch5 ( *m/z* 364); (G) Ch6 ( *m/z* 366); (H) Ch7 ( *m/z* 350)


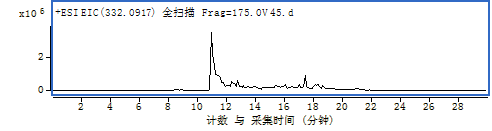

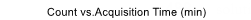


（A）

SA


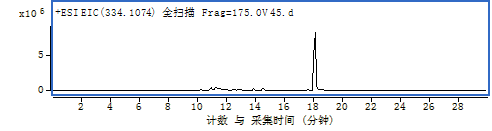

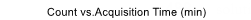

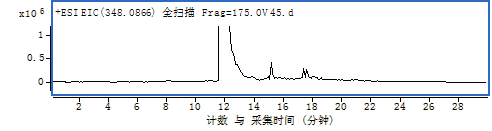

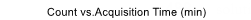

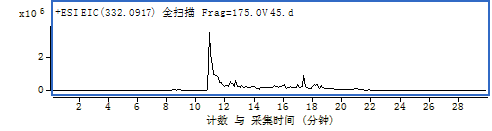

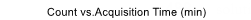


S1

（C）

S2

（B）

（D）

SA


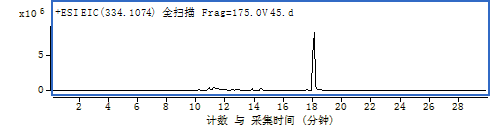

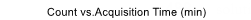


S1

（E）


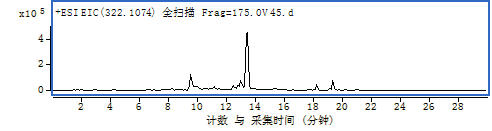

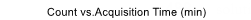


S3

（F）

**Supplementary Figure S-2.** The accurate EIC of SA metabolites: (A) SA (*m/z* 332); (B) S1 (*m/z* 334); (C)S2 (*m/z* 348) in female SD rat feces at 0-24 h; (D) SA (*m/z* 332); (E) S1 (*m/z* 334); (F) S3 ( *m/z* 322) in male SD rat feces at 0-24 h
